# Perceptual Grouping and Visual Enumeration

**DOI:** 10.1371/journal.pone.0050862

**Published:** 2012-11-30

**Authors:** Veronica Mazza, Alfonso Caramazza

**Affiliations:** 1 Center for Mind/Brain Sciences (CIMeC), University of Trento, Rovereto, Italy; 2 Department of Cognitive Sciences and Education, University of Trento, Rovereto, Italy; 3 Department of Psychology, Harvard University, Cambridge, Massachusetts, United States of America; University of Sydney, Australia

## Abstract

We used lateralized Event-Related Potential (ERP) measures – the N2pc and CDA/SPCN components – to assess the role of grouping by target similarity during enumeration. Participants saw a variable number (0, 1, 2 or 3) of same- or differently-colored targets presented among homogeneous distracters, and performed an enumeration task. Results showed that the N2pc, but not the CDA, was larger for multiple targets of identical color relative to targets of different colors. The findings are interpreted in terms of the effects of grouping on early versus late stages of multiple object processing. Within this framework, they reveal that grouping has an effect on early individuation mechanisms, while later processing mechanisms are less prone to such an influence.

## Introduction

Humans are constantly confronted with the need to elaborate several objects simultaneously for various purposes, such as tracking of moving objects and enumeration. The ability to analyze objects as distinct entities is a crucial function of the visual system, and it is accorded a prominent role in several models of high-level vision (e.g., [1], [2]). According to these models, there are at least two separate classes of mechanisms involved in object analysis [2], [3]. Early individuation mechanisms provide a coarse representation of the objects in the visual field, allowing the visual system to individuate each object as being separate from others. While earlier proposals argued that such mechanisms operate in the absence of attention, recent research has suggested that simultaneous indexing of relevant items is tightly related to attention, being indeed one of its key functions [4]. Further support for this idea is provided by recent studies [5], [6], [7], which have found that the rapid and accurate apprehension of small numerosities (the so-called subitizing phenomenon, see [8]) requires attention and does not occur automatically. Later processing mechanisms, most likely involving the operation of visual Working Memory (WM), encode the individuated objects in greater detail, ultimately leading to full recognition and identification.

The proposed distinction between early individuation and late WM-related operations in multiple object processing has been supported experimentally by behavioral measures and to some extent by neuroimaging data. For instance, functional Magnetic Resonance Imaging (fMRI) studies have indicated the existence of two spatially distinct patterns of neural activity (mainly in parietal and occipital extrastriate areas) that seem to correlate with the functional difference between individuation and later processes associated with full object recognition. Crucially, these patterns are modulated by object numerosity, and reach an asymptote at about 3–4 elements, in line with the capacity limit proposed by behavioral models of multiple object processing (for a review, see [9]).

Within the ERP domain, previous studies have found two temporally distinct brain activations during visual tasks in which a single lateralized target is presented together with distracters [10], [11], [12], [13]. A lateralized response at posterior electrode sites (N2pc, 180–300 ms; [14], [15], [16]) is elicited whenever a relevant object is presented in the visual field. A later sustained lateralized activity (Contralateral Delayed Activity, CDA, 350–600 ms [17]; also called Sustained Posterior Contralateral Negativity, SPCN [10]) occurs when the task requires the relevant object to be encoded in greater detail.

The N2pc is generally considered the correlate of attention orienting in the visual field. This component is elicited when participants are required to orient to an object and not when merely required to orient to a location [18], suggesting that it reflects a feature-to-location binding mechanism through which a potentially relevant object is selected from distracters [14]. Recent research [19], [20], [21], [22] has additionally shown that the amplitude of the N2pc is sensitive to the number of target elements presented in various tasks (i.e., multiple object tracking and enumeration) and reaches a plateau at approximately 3–4 elements, in line with the capacity limit proposed by models of object individuation (e.g., [1]). The CDA/SPCN is also modulated by the number of targets and reaches a similar asymptote during the execution of a variety of tasks, including short-term memory, multiple object tracking, and enumeration tasks [17], [19], [22]. Overall, the findings obtained thus far indicate that the N2pc and CDA/SPCN are two neural patterns tightly correlated, respectively, with the object individuation mechanism and with the maintenance of the individuated objects for subsequent cognitive operations required for computing more detailed representations of the objects. In the present study, we used these two neural ERP patterns to explore the role of grouping in exact enumeration of small target numerosities presented together with irrelevant elements (i.e., distracting objects).

The ability to enumerate target objects in a cluttered scene involves at least two stages of analysis. First, the visual system needs to isolate the elements to be counted both from distracters and from each other [23]. An object individuation mechanism that provides a set of representations of space-property bound features, making them ready for further processing, is well suited for this purpose. Second, once these representations are formed, and at least when the elements to be counted are presented briefly, they need to be maintained active in a VWM buffer during the process of mapping the set of selected elements onto a specific numerical value. Probing how grouping influences these two stages provides an opportunity to enrich our understanding of how exact enumeration is achieved in the visual modality.

By definition, grouping involves a relationship among several objects, and it is thus tightly related to how multiple targets are elaborated during enumeration. Since the seminal observations of Gestalt psychology, decades of research have indicated grouping as one of the most powerful factors that govern our perception of the external world (for a review, [24], [25]), although its effects seem to depend on the nature of the task. Indeed, while some studies on multiple object tracking show superior performance when the targets are grouped together (e.g., [26]), others underline the negative effects of grouping. For instance, studies on numerosity estimation have shown that grouping by spatial factors such as connectedness can lead to underestimation of the number of target elements [27]. Studies of the impact of grouping on exact enumeration of small sets of objects have produced contrasting results. Some studies [28], [23] found that homogenous elements (e.g., object sets of the same color) led to lower RTs than heterogeneous targets (e.g., object sets composed of two colors) during subitizing. Other studies have found either the opposite effect [29], although this was mostly visible for larger numerosity sets (i.e., more than 5 objects), or no effect at all [30].

On the whole, then, the role of perceptual grouping on subitizing remains unresolved. Furthermore, since behavioral measures alone may prove insufficient to evaluate the impact of target grouping on the different subcomponents involved in computing object quantity, it would be useful to use ERP measures to address whether perceptual grouping affects early or late cognitive mechanisms involved in exact enumeration of small sets of objects. Here we used the N2pc and CDA/SPCN responses to determine the level(s) of representation that is affected by target grouping when participants enumerate target sets varying in color similarity (same color versus different colors). As discussed above, these components of the EEG signal are modulated by the number of target objects (reaching an asymptote at 3–4 elements) in enumeration tasks, and are interpreted as the electrophysiological counterparts of respectively the individuation mechanism and the WM maintenance of the individuated objects [20], [21], [22]. We expect that if perceptual grouping affects early individuation stages, the N2pc component should be modulated by target similarity. Similarly, if perceptual grouping affects the maintenance of the individuated objects, as some previous studies on the effect of object complexity on memory tasks have suggested [31], [32], [33], [34], [35] we would expect this to be reflected in modulation of the CDA/SPCN.

## General Methods

### Participants

Twenty-four volunteers (mean age 24.3 years) participated in the experiment. Participants provided written informed consent. The experiment was approved by the University of Trento Ethics Committee.

### Stimuli and Procedure

**Figure 1:**
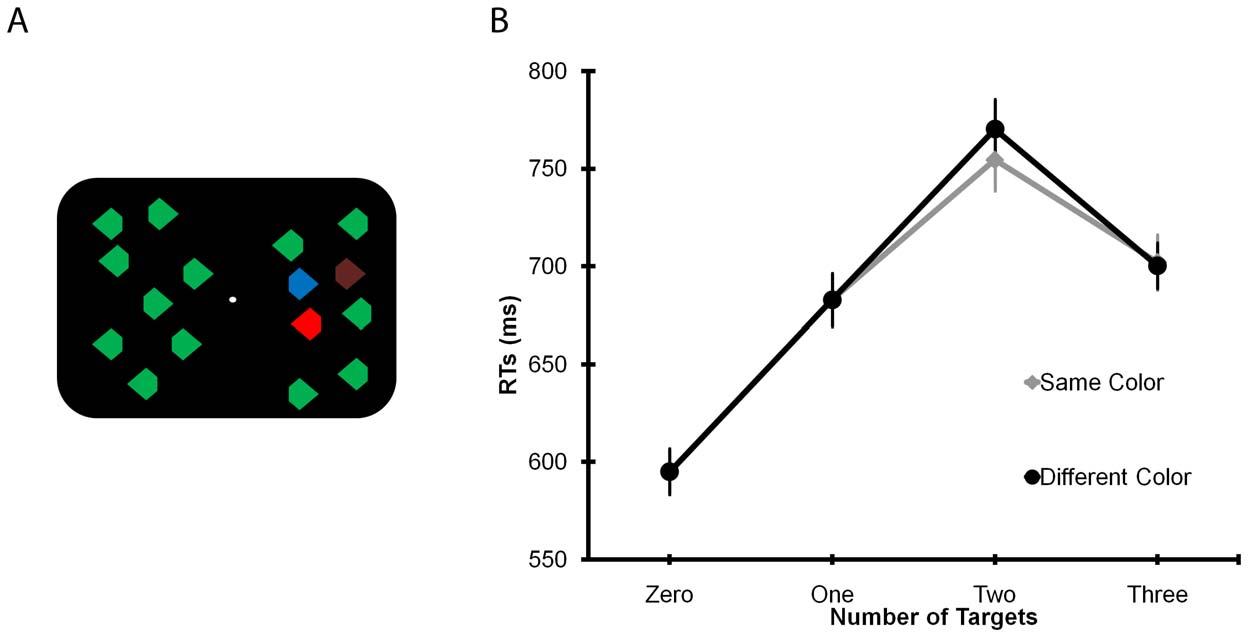
Stimuli and behavioral results. (**A**) Example of a trial with three ungrouped targets on the right visual hemifield. (**B**) Mean RTs (with standard errors) as a function of target numerosity and similarity.

Equiluminant red, green, brown and blue diamonds (17 cd/m^2^) were presented on a black background (1 cd/m^2^). Each diamond (0.6°×0.8°) had a 0.4° corner trimmed on the left or right side (Figure 1a). On each trial, the display contained a total of 16 diamonds, equally distributed to the left and right side of the fixation circle (0.2°). The diamonds were located within a 10 (columns, 11.4°)×8 (rows, 8.6°) matrix. On 1/4 of the trials all diamonds had the same color (zero-target condition). On the other trials, one, two or three diamonds (the targets) had a unique color relative to distracters (either red, green, brown or blue) and appeared with equal probability and in random order to the left or right of fixation, but never in the two columns of the matrix closest to fixation or in the most peripheral columns and rows. Target stimuli were always presented together in the same side, either the left or the right hemifield. On half of the trials with either two or three targets their color was identical (e.g., all targets were green) while on remaining trials they all had different colors (e.g., one target was red and one was green). Zero- and one-target trials (where no effect of grouping can be assessed) were included in the study for two reasons. First, zero-target trials helped reinforce the use of a common “strategy” for selecting the targets (i.e., looking for the color different from the distracter elements) in both the same and different color conditions. Second, one-target trials were useful in order to have a continuum of small numerosities to make the enumeration task more realistic. The color of the target(s) and of the distracters was counterbalanced across participants. Each visual display was presented for 150 ms.

Participants reported as fast as possible the number of targets presented on each trial by pressing one of four keys on a computer keyboard with their index or middle finger of both hands. Response assignment was counterbalanced across participants. Maximum time for responding was 1500 ms. The response to stimulus interval was 1500 ms. Participants performed 9 experimental blocks of 96 trials each.

### EEG Recording and Data Analysis

EEG was recorded from 25 Ag/AgCl electrodes (including PO7, PO8, O1 and O2) and from a left earlobe electrode, with a right-earlobe reference, and then re-referenced offline to the average of the left and right earlobe sites (bandpass filter: 0.01–40 Hz, A/D rate: 1000 Hz). Horizontal EOG (HEOG) was recorded from electrodes positioned on the outer canthi of both eyes. Impedance was kept below 6 kΩ for all electrodes. Trials with horizontal eye movements (HEOG exceeding±30 µV), eye blinks and other artifacts (any electrode exceeding±80 µV) were excluded. The average number of trials retained was 88%. Averages for correct responses were computed for a 700-ms interval starting 100 ms before the display onset, separately for each condition. For target-present trials, we derived the lateralized activations by computing the mean difference amplitudes obtained by subtracting ERP waveforms at posterior PO7/8 and O1/2 electrodes ipsilateral with respect to target location (i.e., PO7 and O1 for left targets) from those recorded at contralateral sites (e.g, PO8 and O2 for left targets), collapsed across target side, for the following post-stimulus intervals: N2pc (180–270 ms), CDA/SPCN (350–600 ms).

The first analysis on both behavioral and ERP data evaluated the effect of color homogeneity by means of ANOVA. Here, zero and one-target trials were excluded, and the factors considered were Color (same versus different), Numerosity (two versus three targets) and Component (N2pc versus CDA; for ERP data only). In a second set of ANOVAs, we assessed the effect of target numerosity separately for the conditions with same and different target color. We could not perform a single ANOVA since for the zero- and one-target trials the factor Color (different versus same) was meaningless. The factors considered were Numerosity (zero, one, two, and three targets for behavioral data; one, two, and three targets for ERP data; data for zero- and one-target trials were the same in both ANOVAs) and Component (N2pc versus CDA, for ERP data only). When appropriate, Greenhouse-Geisser correction for sphericity violations was applied, and only the corrected p values are reported. Further analyses were conducted by means of pairwise *t*-tests and contrast analysis (for the factor Numerosity when involving more than 2 levels).

## Results

### Behavioral Performance

Three separate ANOVAs were conducted respectively on response times (RTs) for correct responses between 200 and 1500 ms, and on correct responses.

### RTs

The ANOVA assessing the effect of color homogeneity (factors: Numerosity: 2, 3; and Color: same, different) showed significant effects of Numerosity, *F*(1,23) = 86.5, *p*<.001, η^2^ = .79, and of Numerosity×Color, *F*(1,23) = 12.7, *p* = .002, η^2^ = .36. Follow-up comparisons (*t*-tests) revealed that participants were slightly faster on two-target trials with same color relative to the different color trials, *t*(23) = 4.8, *p*<.001, while no difference emerged for three targets (*p* = .77; see Figure 1b).

The ANOVAs evaluating the effect of target numerosity (factor: Numerosity: 0, 1, 2, 3 targets) in the same and different color conditions showed a significant effect of Numerosity for both conditions (same color: *F*(3,69) = 76.0, *p*<.001, η^2^ = .77; different color: *F*(3,69) = 106.9, *p*<.001, η^2^ = .82), with an increase in RTs up to two targets, and a decrease for three targets, as confirmed by a significant quadratic effect in the contrast analysis (same color: *F*(1,23) = 139.1, *p*<.001; different color: *F*(1,23) = 154.3, *p*<.001). This pattern reflects the so-called end effect, in which participants are faster and more accurate for the extreme values of a given numerosity set (i.e., zero and three in the this experiment).

### Accuracy

Participants’ accuracy was quite high (>90% correct) overall. The ANOVA for the effect of color homogeneity (factors: Numerosity: 2, 3; and Color: same, different) showed significant effects of Numerosity, *F*(1,23) = 29.6, *p*<.001, η^2^ = .56, and of Numerosity×Color, *F*(1,23) = 5.2, *p* = .03, η^2^ = .18, but no significant difference was found between same and different colors for any numerosity, all *p*s≥.1.

The ANOVAs evaluating the effect of target numerosity (factor: Numerosity: 0, 1, 2, 3 targets) in the same and different color conditions showed a significant effect of Numerosity for both conditions (same color: *F*(3,69) = 35.1, *p*<.001, η^2^ = .6; different color: *F*(3,69) = 26.3, *p*<.001, η^2^ = .53), with a quadratic trend similar to those for RTs (zero target: *M* = 99%; one target: *M* = 96%; two targets same color: *M* = 91%; two targets different color: *M* = 91%; three targets same color: *M* = 96%; three targets different color: *M* = 93%; same color: *F*(1,23) = 69.0, *p*<.001; different color: *F*(1,23) = 23.7, *p*<.001).

### ERP Results

**Figure 2:**
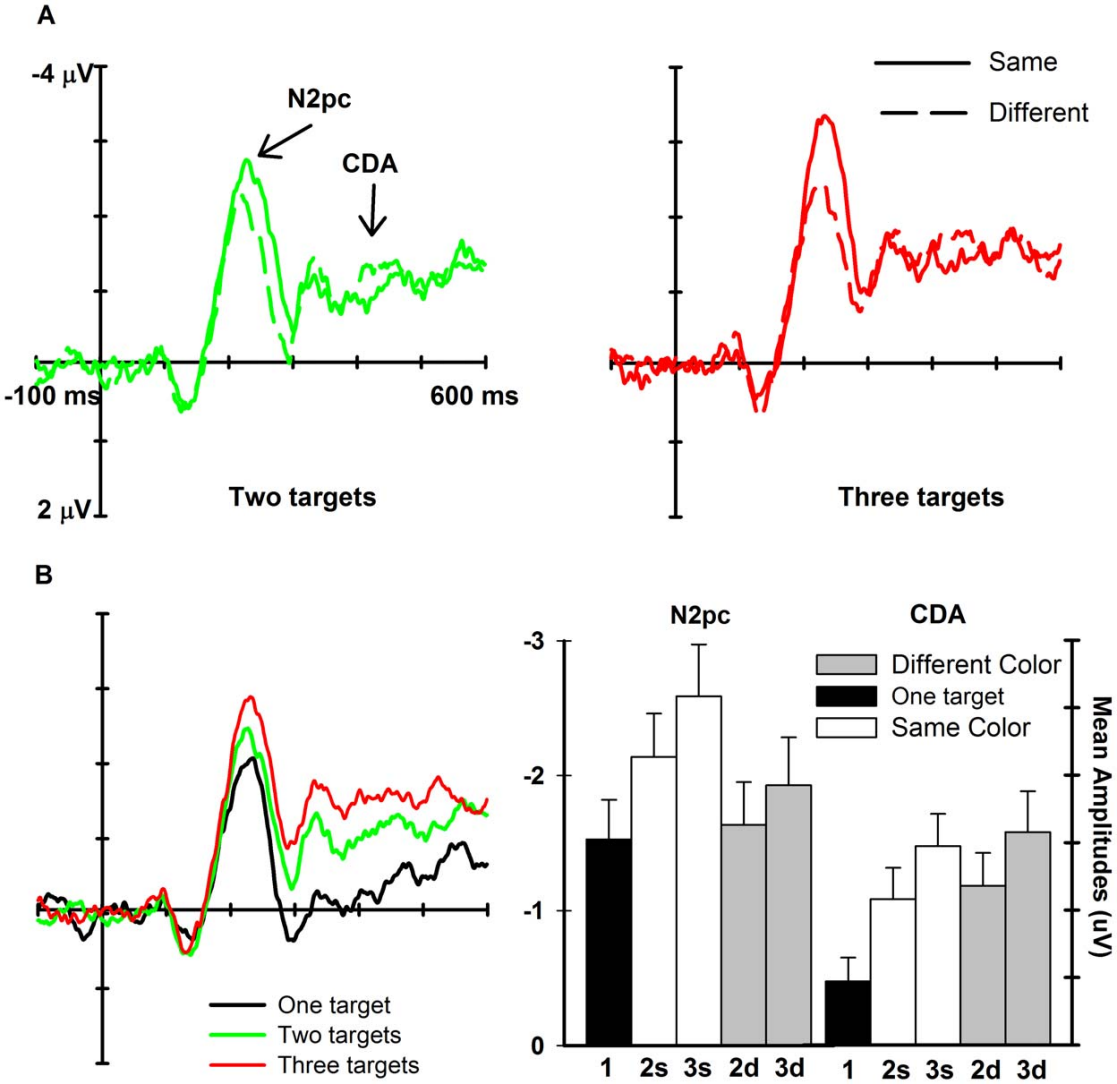
ERP results. (**A**) The grand-average ERP difference waveforms show the larger N2pc on same relative to different color trials, both for two (left side) and three (right side) targets. Difference waveforms were obtained by subtracting ipsilateral activations from contralateral activations recorded at posterior sites (PO7, PO8, O1 and O2), collapsed across target side. (**B**) Left: The grand-average ERP difference waveforms, collapsed across same and different color trials, show the larger N2pc for larger numerosities. Right: N2pc and CDA mean amplitudes (with standard errors) as a function of target numerosity both in the same (white) and different (grey) color conditions. Amplitudes for one-target trials are depicted in black.

The N2pc was larger for the same-color than the different-color condition; there was no effect of color similarity on the CDA/SPCN (Figure 2a). By contrast, the N2pc and CDA/SPCN amplitudes increased as a function of target numerosity in both the same- and different-color conditions (Figure 2b). Statistical analyses confirmed these observations.

The ANOVA assessing the effect of color homogeneity (factors: Numerosity: 2, 3, Color: same, different; and Component: N2pc, CDA) showed a significant effect of Numerosity, *F*(1,23) = 16.3, *p* = .001, η^2^ = .41, with three targets eliciting more pronounced N2pc and CDA amplitudes relative to two targets. The effects of Color and Component were also significant (*F*(1,23) = 7.7, *p* = .01, η^2^ = .25, *F*(1,23) = 14.5, *p*<.001, η^2^ = .39, respectively). Importantly, the Color×Component interaction was also significant, *F*(1,23) = 33.1, *p*<.001, η^2^ = .59. Separate ANOVAs exploring this interaction revealed more pronounced N2pc amplitudes for the same – than for the different – color condition, *F*(1,23) = 32.2, *p*<.001. In contrast, color similarity was not significant for the CDA (*F*<1).

The ANOVA assessing the effect of target numerosity for the same-color condition (factors: Numerosity: 1, 2, 3; and Component: N2pc, CDA) showed a significant effect of Component, *F*(1,23) = 21.6, *p*<.001, η^2^ = .48 and importantly of Numerosity, *F*(2,46) = 19.6, *p*<.001, η^2^ = .46. The contrast analysis exploring the effect of Numerosity revealed a significant linear trend, *F*(1,23) = 37.6, *p*<.001, indicating larger N2pc and CDA amplitudes for larger numerosities.

The ANOVA for the different-color condition (factors: Numerosity: 1, 2, 3; and Component: N2pc, CDA) indicated significant effects of Component, *F*(1,23) = 11.9, *p* = .002, η^2^ = .34, Numerosity, *F*(2,46) = 11.5, *p*<.001, η^2^ = .33, and Component×Numerosity, *F*(2,46) = 13.3, *p*<.001, η^2^ = .37. However, separate ANOVAs exploring this interaction revealed a significant effect of Numerosity for both the N2pc and CDA (N2pc: *F*(2,46) = 4.54, *p* = .02; CDA: *F*(2,46) = 17.6, *p*<.001), with the contrast analysis indicating a linear trend in both conditions (N2pc: *F*(1,23) = 6.0, *p* = 02; CDA: *F*(1,23) = 20.7, *p*<.001).

### N2pc Peak Amplitudes

Additional analyses were conducted on the N2pc peak amplitudes (quantified as the maximum negative difference amplitudes in the 150–350 ms post-stimulus interval for each condition). No such analysis was carried out on the CDA as no clear peak can be measured for this component. In line with the main analyses, the ANOVA assessing the effect of color homogeneity (in which only the two- and three- target trials could be included) showed significant effects of color, with more pronounced N2pc peak amplitudes for the same- than for the different-color condition, and also a significant effect of numerosity, with three targets eliciting a more pronounced N2pc relative to two targets, both Fs>21.0, both ps<.001. The ANOVAs assessing the effect of target numerosity for both the same- and different-color conditions showed a significant effect of numerosity (same color: F(2, 46) = 25.1, p<.001; different color: F(2, 46) = 10.37, p<.001). A significant linear trend emerged in both conditions, both Fs>12.3, both ps<.003, indicating larger N2pc amplitudes for larger numerosities.

### Control Analysis

Under the assumption that sensory properties should influence the earliest stages of stimulus processing, such as the one reflected by the P1 component, we measured this ERP response to control for the presence of sensory effects related to the increase in target numerosity and to color similarity. We conducted three additional ANOVAs on the P1 mean amplitudes (60–100 ms post-stimulus) with the same factors used for the N2pc and CDA components. No significant effect emerged, suggesting that the earliest stages of stimulus processing are not influenced by these factors.

## Discussion

In this study we tested the effect of perceptual grouping on exact enumeration of small quantities of objects. According to a two-stage model of object analysis [1], [2] both early individuation/selection mechanisms and late WM-related operations are involved in simultaneous processing of multiple objects in various tasks, including the enumeration task used in the present study. Our results suggest that the impact of grouping is located at the early individuation stage.

When response times for two targets were considered, we found that participants were slightly faster for the same color condition. While this aspect is suggestive of facilitatory effects of grouping on subitizing, it was found only for numerosity two and thus requires further investigation to evaluate its robustness. The ERP results provided clearer evidence of the influence of perceptual grouping on target enumeration, and in particular on the specific stage of processing affected by grouping. The ERP results revealed different effects of perceptual grouping for early and late stages of multiple object processing. We found that the N2pc was clearly modulated by target similarity, being overall larger for multiple targets with identical color. The N2pc was also larger for the larger target sets for both same- and different- colored targets. By contrast, while the CDA/SPCN component was also clearly modulated by target numerosity, there was no effect of visual similarity on this ERP component.

One may wonder about the extent to which the effects found in our study are related to enumeration per se. For instance, the results might be interpreted in terms of the amount of “difference” detected in a region of the display. However, as previously shown ([21], Experiment 2), differences in a specific part of the display cannot directly account for the N2pc numerosity-related modulations. In that study, 0, 1, 2 or 3 targets were presented in a different color from distracters, and participants were asked to report whether at least one target was present in the display. Results indicated no modulation of the N2pc as a function of target numerosity. This result reasonably argues against an interpretation that sees the variation in N2pc found in the current study as a simple reflection of the amount of difference detected in a region of the display. Overall, and in line with previous theoretical models [1], we propose that the N2pc (and its variations) reflects the operation of a general individuation mechanism that is at work not only for the specific case of exact enumeration, but also in other contexts that require multiple object selection, such as for instance object tracking [19].

Alternatively, the results could reflect variations in sensory parameters, such as the relative area occupied by the targets. We acknowledge that this may be a possible account of the overall N2pc numerosity-related modulations found in the present data. However, it is not clear why this should differentially affect homogenous and heterogeneous target trials – the main manipulation in the present study. In our study, the targets were made equiluminant in both same and different color conditions and the increase in the relative area occupied by the target was the same in both conditions. Moreover, under the assumption that sensory properties should influence the earliest stages of stimulus processing, such as the one reflected by the P1 component, we conducted an additional analysis on this ERP response, but found no effects of color or numerosity. Therefore, it is difficult to explain the N2pc pattern found in the present study directly and exclusively in terms of sensory effects, although we acknowledge that future research will need to address this issue more deeply. Finally, a previous study by Drew and Vogel [19] directly addressed this issue, and found no effect of area on the N2pc and CDA, suggesting that the spatial extent of the target area cannot be uniquely responsible for the present effects.

Taken together, our results provide new information on the way multiple object processing is accomplished. According to previous research and theoretical models, object individuation is based on spatio/temporal information alone [3]. However, these proposals cannot account for situations that are very common in everyday life, wherein only some objects out of the many items present in the environment are relevant for a given goal. The N2pc modulation as a function of target numerosity found in the present study in the same- and different-color conditions indicates that the representations formed in the neural structures generating this ERP response are not feature-blind, but encode both spatial and non-spatial feature information. Therefore, these data provide an extension of previous individuation models by pointing to the potential perceptual locus where feature-to-location binding takes place for full object individuation. These results also have significant implications for theories of object-number mapping, suggesting that the representations generated by the neural structures underlying the N2pc contain the fine-grained information required for exact enumeration: namely, the ability to extract the precise numerosity of the relevant objects when presented together with irrelevant ones, both when they possess the same unique feature and when they are different from each other.

The N2pc data reported here additionally highlight that the individuation process is modulated by target similarity, with overall larger amplitudes for targets with identical color. Models of attention selection [36] have proposed the existence of a priority map , in which some elements receive preferential processing by virtue of either their physical properties (e.g, because they possess a unique color with respect to the other elements) or task relevance. For instance, it has been shown that a salient object, such as one possessing a higher value in a specific feature dimension relative to the other objects, can get prioritized even when it is task irrelevant [37], but see [38]. While in our study the targets’ colors were equiluminant, thus excluding a direct account of the data in terms of physical salience, it is nonetheless possible that the overall level of targets’ priority was enhanced as a consequence of grouping by color. This would be consistent with previous research [39] indicating that target grouping by color improves the ability to judge the global shape formed by the target elements. More generally, the present results suggest that the priority map accumulates evidence for multiple locations/items, and that grouping in the color domain can change the priority given to a set of target elements.

Interestingly, recent behavioral research on enumeration [40] has shown that the relative salience of one target with respect to the others can reduce the subitizing range. However, when the global salience of the target elements is considered (i.e., when the salience of the entire group of targets is manipulated, see [41]) the reduction in the capacity limit is no longer present. In line with these latter results, the absence of a significant interaction between target numerosity and similarity in the N2pc data suggests that grouping does not substantially change the capacity limit of the individuation mechanism in the process of enumeration, at least when the quantity set is well within the subitizing range.

In contrast with the pattern of modulation found for the N2pc, the CDA/SPCN was virtually identical for the same- and different-color conditions, being only modulated by target numerosity. Previous ERP research on visual memory tasks has found that the CDA/SPCN is modulated by target complexity. For instance, it has been shown [32], [34] that when complex as opposed to simple shapes are presented, the CDA amplitudes no longer increase beyond two items. This suggests that the maintenance of fine-grained information about objects reduces the capacity of WM during a memory task. Similarly, fMRI research [35] has shown specific effects in the Intraparietal Sulcus (IPS) related to targets having the same versus different shapes. In the light of these previous results, and in line with a recent study [42] showing no effect of the overall stimulus contrast on the CDA during a delayed match-to-sample task, our findings suggest that enumeration of a small number of items does not require the maintenance of the fine-grained information required to perform a memory task. This may be specifically valid in the context of our study in which the task required participants to count the number of items that were of a different color relative to distracters, without having to encode their specific features.

In conclusion, the present study demonstrates that perceptual grouping has specific effects on multiple object processing in an enumeration task. Our data indicate that grouping influences the individuation mechanism stage, possibly modulating the overall level of priority of a grouped set of potentially relevant objects. By contrast, no effect of grouping is visible at later stages of processing, suggesting that enumeration of small sets of targets does not require the maintenance of specific information related to the target features.
